# Vaccine approaches for the 'therapeutic management' of *Mycobacterium avium* subspecies *paratuberculosis* infection in domestic livestock

**DOI:** 10.1080/01652176.2019.1667042

**Published:** 2019-09-16

**Authors:** Saurabh Gupta, Shoor Vir Singh, Manju Singh, Kundan Kumar Chaubey, Kumaragurubaran Karthik, A. K. Bhatia, Naveen Kumar, Kuldeep Dhama

**Affiliations:** a Department of Biotechnology, GLA University, Mathura, Uttar Pradesh, India;; b Central University Laboratory, Tamil Nadu Veterinary and Animal Sciences University, Chennai, Tamil Nadu, India;; c Veterinary Type Culture Collection, NRC on Equines, Indian Council of Agricultural Research, Hisar, Haryana, India;; d Division of Pathology, ICAR-Indian Veterinary Research Institute, Izatnagar, Bareilly, Uttar Pradesh, India

**Keywords:** *Mycobacterium paratuberculosis*, Johne’s disease, vaccine, livestock, production

## Abstract

High endemicity of Johne’s disease (JD) in herds adversely affects heavy milk yielding breeds by reducing the per animal productivity and ‘productive life-span’. This review evaluates different vaccines used for its control and summarizes the benefits of ‘global vaccine’ in the four major domestic livestock species, namely goat, sheep, buffalo and cattle. Vaccines developed by using ‘native strains’ revealed both 'therapeutic' and preventive effects in domestic livestock. The 'therapeutic' role of vaccine in animals suffering from clinical JD turned out to be valuable in some cases by reversing the disease process and animals returning back to health and production. Good herd management, improved hygiene, ‘test and cull’ methodology, proper disposal of animal excreta and monitoring of MAP bio-load were also regarded as crucial in the 'therapeutic' management of JD. Vaccine approaches have been widely adopted in JD control programs and may be considered as a valuable adjunct in order to utilize huge populations of otherwise un-productive livestock. It has been shown that vaccination was the preeminent strategy to control JD, because it yielded approximately 3–4 times better benefit-to-cost ratios than other strategies. Internationally, 146 vaccine trials/studies have been conducted in different countries for the control of JD and have shown remarkable reduction in its national prevalence. It is concluded that for JD, there cannot be global vaccines or diagnostic kits as solutions have to come from locally prevalent strains of MAP. Despite some limitations, vaccines might still be an effective strategy to reduce or eradicate JD.

## 1. Introduction

Johne’s disease (JD) caused by *Mycobacterium avium* subspecies *paratuberculosis* (MAP) affects domestic livestock population world-wide (Ayele et al. 2001; Chaubey et al. 2016). Bio-load of MAP in the Indian domestic livestock population has shown increasing trend in last 28 years (Singh et al. 2014a). Of the four domestic livestock species in India, bio-load of MAP has been reported to be highest (16.0–54.7%) in the sheep population, followed by 28.3–48.0% in buffaloes, 6.0–39.3% in cattle and 9.4–20.1% in goats (Kumar et al. 2008; Yadav et al. 2008; Sharma et al. 2008; Singh et al. 2014a; Mukartal et al. 2016).

Once this disease enters any herd it becomes established and endemic, since MAP is passed from one generation to another through semen, during pregnancy, by feeding of milk and colostrum and by oral-fecal route from contaminated environment (Buergelt et al. 2006; van Roermund et al. 2007; Eisenberg et al. 2010) and enters the human food chain leading to potential public health issues (Chaubey et al. 2017). In severely affected herds, losses are difficult to estimate since animals get culled early on health and production grounds from the elite germ-plasm developed through many years of genetic selection and breed improvement programs. Despite very high slaughter rate of domestic livestock (goats, sheep and buffaloes) to meet the ever growing demand of meat for domestic consumption and export, bio-incidence of JD continues to increase and has become endemic in several herds in the country. In addition, ‘test and cull’ is not an economically viable option for third world countries like India. Furthermore, ‘test and cull’ methodology has not yielded assured results in goats, though still practiced to reduce environmental contamination by removing shedder goats (Singh et al. 2014a). Similar findings have been reported in other parts of the world with respect to goats (Munir et al. 2014), buffaloes and cattle (Kirkeby et al. 2016; Konboon et al. 2018), where 'test and cull' methodology has been in use for long time but was found to be in-effective, therefore switched to vaccination for the control of the disease. Combined approaches using vaccination with ‘test and cull’ was far more economical and more effective strategy to control persistent losses and disease incidence in various herds of goats, buffaloes and cattle (Dorshorst et al. 2006; Kirkeby et al. 2016).

This review paper summarizes indigenous and global vaccines and vaccination approaches currently used in order to control JD with respect to improvements in the body condition, health, productivity and other parameters in the four major domestic livestock species, considering there is ban on cow slaughter in India.

### 1.1. Options for the control of JD

JD may be controlled by preventing newer cases of infections in calves or by eliminating source of infection, which can be achieved by identifying infected sub-clinical and clinical shedder animals and then either culling or segregating them from the healthy animals/stock (Kirkeby et al. 2016). In developing and poor countries where disease is highly endemic, it is not possible to indiscriminately cull large number of infected animals with high to very high level of infection (super shedders), primarily due to economic reasons. Therefore, it will be prudent to first focus on culling or segregation of super-shedders and then focus should be on resistant/resilient animals known to have received an infectious dose of MAP bacilli at an age when they were susceptible but not infected or remains in a dormant state so that when the animal is examined at necropsy, the infection cannot be detected by culture of tissues and there is no evidence of disease in the histopathological examination also (Whitlock et al. 2005; Whittington et al. 2017).

In view of the chronic and insidious nature of the disease, control programs can be time consuming and may take a minimum of 5 years or longer to be successful in controlling JD. Countries without paratuberculosis control practices of any kind are likely to suffer with greatest impact to human welfare through reduced production of animal protein and potential zoonotic impact. The practices and tools for the control of JD are well known and predominantly limited to breaking the transmission cycle. Culling (forced removal) of clinical cases, 'test and cull' approach for sub-clinical cases, hygienic rearing of young animals, bio-security measures and management of shed environment and pasture were leading approaches. Shed complexes and calving pens should be cleaned to reduce fecal contamination on the coats of animals. Weaning of the calves after colostrum feeding from JD-negative dams helps in reducing the risk of infection to new animals from infected parents. In already infected herds, manure management associated with feeding of colostrum or milk from JD-negative dams should get priority. Water should be piped and ponds and streams should be fenced to minimize fecal contamination of drinking troughs and grazing area may help to reduce losses due to JD (NADIS 2009). These precautions are not practiced and grossly over-looked in livestock farming in India, since majority of the livestock population of the country is in the hands of poor and marginal farmers. As a consequence, livestock frequently graze on public properties and is categorized as 'zero-input agriculture', with little or no attention on health care and lack of additional inputs in the form of green fodder and concentrates.

### 1.2. Test and cull methodology

MAP infection is predominantly prevented by closing new animal additions or securing additions or replacements from JD-free/negative herds (Kirkeby et al. 2016). Off-springs of positive cows are at risk for infection and should be either segregated or tested biannually in case of goats and sheep (Munir et al. 2014) and annually in case of cattle and buffaloes (Kirkeby et al. 2016; Konboon et al. 2018). For the elimination of JD, culling of daughters of sero-positive or culture-positive cows has been practiced in some of the developed countries. Annual testing of adult animals in herds is essential to identify and cull asymptomatic, sub-clinical and clinical shedders. Application of the diagnostic tests in the control programs has been critical for the chronic, insidious and spectral disease like JD, with focus on the presence and absence of bacilli and/or antibodies (Chaubey et al. 2016). Though culture is more sensitive and considered ‘Gold standard’ test ELISA has been found to be quick and cost effective as screening test (Gupta et al. 2017). Problem with this approach is the long and variable time interval between the infection and time when the animals will either test positive or exhibit clinical symptoms (incubation), which in turn is dependent mainly on factors like management, nutrition, health care and other in-puts.

### 1.3. Vaccination strategy

Vaccination is the most efficient and cost-effective strategy for the prevention of appearance of the clinical cases in herds. Only seven countries have a control program in place that include vaccination. Major reason for not using vaccine is likely due to the interference of JD vaccines with serological tests for bovine tuberculosis (Coad et al. 2013; Serrano et al. 2017). Control of JD using vaccination, ‘test and cull’ or combined approaches was economical and has been used as a tool to aid the control programs for JD in Australia, New Zealand, Spain, the Netherlands, Canada, Iceland and India (Basida and Juste 2011; Singh et al. 2015; Shephard et al. 2016; Whittington et al. 2019). However, vaccination has been prohibited in Denmark, Norway and Sweden and stamping out have been used as control practices instead. As an example, Australia used vaccine approach (5–35 years) to reduce infection from >35% to <1% (Hore et al. 1971; Dhand et al. 2016; Whittington et al. 2019). Now USA is also adopting a mutant vaccine approach in controlling bovine JD. Control by vaccination provided minimal long-term losses with cost effective control over 10 year planning horizon (Lamont et al. 2014) (Table 1). In India, vaccination using ‘native strain’ administered to domestic livestock has improved per animal productivity and helped to conserve the threatened native breeds of domestic livestock, specially milk breeds due to JD in Indian conditions (Singh et al. 2017; Whittington et al. 2019). Recently, ELISA-based tests ('indigenous ELISA using whole cell protoplasmic antigens and recombinant secretory proteins based cocktail ELISA) were developed Indigenously to differentiate between infected and vaccinated animals (DIVA), since animals are not numbered in India and vaccine program may interfere in JD surveillance program (Chaubey et al. 2018).

**Table 1.** Transposon mutant vaccine candidates of MAP.

Institution^a^	Location of insertion^b^	MAP strain^c^	References
USDA-ARS-WRRC	MAP0482	Goat strain 43432-02	McGarvey, unpublished
University of Wisconsin	MAP3006c (*lipN*)	K-10	Bannantine, 2014
Washington State University	MAP1047 (*relA*)	K-10	Park et al. 2014
University of Nebraska	MAP1566	K-10	Rathnaiah et al. 2014
University of Nebraska	MAP3695 and *fadE5*	K-10	Rathnaiah et al. 2014
University of Nebraska	MAP0460 (*lsr2*)	K-10	Rathnaiah et al. 2014
University of Nebraska	MAP0282c and 0283c	K-10	Rathnaiah et al. 2014
University of Nebraska	MAP1566	K-10	Rathnaiah et al. 2014
University of Nebraska	MAP2296c and 2297c	K-10	Rathnaiah et al. 2014
University of Nebraska	MAP1150c and 1151c	K-10	Rathnaiah et al. 2014
New York, USA	*leuD*	Strain 66115-98	Faisal et al. 2013
University of Wisconsin	MAP1872c (*mbtH_2*)	ATCC19698	Kabara and Coussens, 2012
AgResearch NZ	MAP1566	strain 989	Scandurra et al. 2010
AgResearch NZ	MAP0011 (*ppiA*)	K-10	Scandurra et al. 2010
Washington State University	MAP3893c (*pknG*)	K-10	Park et al. 2011
Washington State University	MAP0460 (*lsr2*)	K-10	Park et al. 2011
University of Wisconsin	MAP3963 (*umaA1*)	ATCC19698	Shin et al. 2006
University of Wisconsin	MAP2408c (*fabG2_2*)	ATCC19698	Shin et al. 2006

a The location of the laboratory where the mutant(s) was constructed.

b The MAP locus where the transposon had been inserted. If two genes are listed, the transposon is inserted in the intergenic region between the two. If the gene has been named, it is shown in parenthesis.

c The parental strain of MAP used to create the mutation.

Several other studies directed toward the development of subunit (such as immunogenic secretory proteins) or vectored vaccines (Faisal et al. 2013; Thakur et al. 2013; Gupta et al. 2016). Although these technologies are still under development and validation, they may provide effective vaccines in near future. Live vaccines may result in reduction of clinical disease in infected herds, but will not lead to eradication of infection. Immunity frequently breaks down when vaccinated animals are sold to other herds, negating the value of vaccination for herds selling breeding animals for replacements. As a result of this and because of vaccination with a live organism that may be capable of potentially infecting humans, therefore live vaccines are not favored by several countries (Park and Yoo 2016). Killed vaccines are preferably used and positive cost benefits have been reported with their use (Table 2). Currently there are limited number of killed vaccines licensed internationally against JD (Windsor 2006; Patton 2011; Singh et al. 2017).

**Table 2.** Commercial Johne’s disease vaccines in the international market.

Sn	Name/kind of vaccine	Vaccine strain and bio-type	Adjuvants	Countries
1.	Fromm	MAP Strain 18, Killed	Oil type (Freund’s complete)	USA
2.	Lio-Johne^a^	316F strain, Live attenuated	Oil type	Spain
3.	Phylaxia	5889 Bergey, Killed	Oil type	Hungary
4.	Weybridge Vaccine	316F strain, Live attenuated	Paraffin and olive oil with pumice stone powder	United Kingdom
5.	Gudair^#^ (Zoetis Pfizer)	MAP Strain 318F, Killed	Oil emulsion	Australia
6.	Aqua Vax Map	Strain 316F, Live attenuated	Water based (saline)	New Zealand
7.	Neoparasec (MerialNZ Ltd.)	Freeze Dried Live MAP, Live attenuated	Oil type	France
8.	Mycopar^#^	Whole cell bacterin, inactivated	Oil emulsion	Germany
9.	Silirum (Pfizer CSL)	MAP Strain 318F, Killed	Oil emulsion	Australia
10.	Bio-JD Oil & Gel (Biovet Pvt. Ltd.)^b^	Native MAP strain ‘S 5’ 'Indian Bison type', Inactivated	Aluminium hydroxide gel (Gudair, Spain), Gerbu adjuvant (Gerbubiotechnik, Germany)	India (2004–2014)*

^a^For sheep and goats.

^b^For goats, sheep, cattle and buffaloes licensed by Drug Controller, Government of India (DCGI, New Delhi, license no. KTK/28D/11/2008) and candidate vaccine strain and technology has been transferred to M/S Biovetpvt. Ltd., Bengaluru, India.

Research into development of improved vaccines is being undertaken in many countries in the world. The main drawback to vaccination is that, since vaccines used in the field are not DIVA and can interfere with serological diagnosis of paratuberculosis and tuberculosis infections. There is also potential for interference with the skin test for tuberculosis. Tests capable of DIVA has been successfully developed and validated using field samples (Chaubey et al. 2018).

Over the period of few years, economic advantages of vaccination might be much higher than 'test and cull' strategy and combined approaches may be most effective in clinical shedders. Furthermore, it has been suggested that vaccination might be the beginning of the end of this devastating problem of domestic livestock world-wide known as ‘paratuberculosis’ and might mark the difference between doing nothing and advancing towards global control (Juste et al. 2002). An ideal JD vaccine should have following properties:

 i. Ideal JD vaccine
  a. Cause minimal tissue injury.
  b. No interference between tuberculosis and paratuberculosis disease diagnosis.
  c. Discriminate between infected and vaccinated animals against paratuberculosis.
  d. Eliminate or reduce fecal shedding of bacteria.

Globally efforts are on to develop various type of vaccines with superior efficacy against JD of domestic livestock.

  ii. JD vaccines under development globally.
   a. Modified live attenuated whole cell MAP vaccines.
   b. Gene knockout whole live MAP vaccines; live mutant vaccine: by random, direct and insertional mutagenesis.
   c. Killed cell wall deficient (CWD) whole cell vaccines.
   d. Vector-based vaccine: use of *M. bovis BCG* as vector to express MAP proteins.
   e. Protein subunit vaccine MAP: recombinant MAP Hsp70, 74F, Ag85AA, g85BAg, 85C, SOD.
   f. DNA vaccine: plasmid DNA encoding cocktail of MAP proteins.

In the global movement against JD, USA (bovine JD) besides Canada (Ovine JD) is latest to join and adopt vaccination program after the moderate success of ‘test and cull’ methodology. Control of disease using combined approach; of 'test and cull' and vaccination was more cost-effective than using ‘test and cull’ and/or vaccination. Internationally, 146 vaccine trials/studies have been widely conducted in different countries for the control of JD and have shown remarkable reduction in the National prevalence of JD (Whittington et al. 2019).

### 1.4. Monitoring parameters for ‘vaccination trials’

Vaccinated animals can be monitored for the response as per the methodology of Johne’s Disease Integrated Program (JDIP) of the USA with some modifications (Singh et al. 2007).

#### 1.4.1. Herd profile

Age (6–12 months, 12–18 months and adult) and sex-wise (males/females) profile of the animals in the herds should be prepared. All the animals in a herd above 3 months of age were vaccinated with ‘vaccine’ as above, irrespective of sex, physical condition, physiological state and health condition (sub-clinical, clinical and advance clinical) with respect to MAP infection.

#### 1.4.2. History of JD

Status of JD was estimated on the basis of history, mortality, morbidity, necropsy, screening of the farms, culling for JD disease infection, etc.

#### 1.4.3. Screening of animals before vaccination

Fecal, serum, blood and milk samples were screened twice at monthly intervals using microscopy, culture, indigenous ELISA kit and IS900 PCR.

#### 1.4.4. Monitoring of vaccinated animals

Vaccinated and control groups were monitored on following parameters from 0 to 360 days post vaccination on the basis of health (mortality, morbidity, etc.), body condition score and production parameters (birth weights, body weights gained, reproductive efficiency, etc.), physical condition (diarrhea, weakness, etc.), immunological parameters (ELISA titer or sero-conversion) and status of shedding of MAP. However, necropsy findings of animals died during the program was conducted and included. Tissues were examined for the presence and absence of gross and microscopic lesions of JD in visceral organs and particularly in mesenteric lymph nodes and intestinal mucosa.

## 2. Comprehensive analysis of effects of JD vaccines world-wide

### 2.1. Effects of vaccination on shedding of MAP bacilli

Screening of fecal samples at different time points (days post vaccination) showed that there was marked reduction (5.8–99.1%) in shedding of MAP bacilli in fecal samples (Table 3). Studies showed vaccination was the pre-eminent strategy to control JD, because it yielded approximately 3–4 times better benefit-to-cost ratios than other strategies (Park and Yoo 2016; Singh et al. 2017). Vaccination improved the immunity of individual animals and was able to arrest the progression of infection, which resulted in reduction of the shedding of bacilli.

**Table 3.** Effects of vaccination on shedding of MAP bacilli (globally).

Sn	Name/kind of vaccine	Country	Species (breed)	Reduction (%)	Period of study	References
1.	Laboratory Scale (Live)	USA	Cattle	81.4	–	Larsen et al. 1974
2.	Fromm (Killed)	USA	Cattle	99.1	–	Hurley et al. 1983
3.	Live attenuated	USA	Cattle	90.0	–	Saxegaard&Fodstad, 1985
4.	Laboratory Scale (Live)	Denmark	Cattle	92.9	–	Jorgensen, 1983
5.	Laboratory Scale (Live)	France	Cattle	81.6	5 years	Argente, 1992
6.	Phylaxia (Killed)	Hungary	Cattle	94.7	–	Kormendy, 1994
7.	Neoparasec (Live)	Germany	Cattle	86.8	–	Klawonnet al. 2002
8.	Lio-Johne (Live)	Spain	Sheep	80.8	–	Aduriz, 1993
9.	Laboratory Scale (Live)	Greece	Sheep	93.2	–	Dimareli-Malli et al. 1997
10.	Gudair (Killed)	Australia	Sheep	90.0	–	Eppleston et al. 2004
11.	Laboratory Scale (In-activated)	India	Goat (Barbari)	82.1	–	Singh et al. 2007
12.	Laboratory Scale (In-activated)^a^	India	Goat (Barbari type)	62.1	7 months	Singh et al. 2010a
13.	Laboratory Scale (In-activated)^a^	India	Goat (Jamunapari)	26.8	7 months	Singh et al. 2013
14.	Laboratory Scale (In-activated)^a^	India	Sheep (Bharat Merino)	27.3	3 years	Singh et al. 2013a
15.	Laboratory Scale (In-activated)^a^	India	Sheep (Patanwadi)	17.1	4 months	Shroff et al. 2013
16.	Laboratory Scale (In-activated)^a^	India	Goat (Mehsana)	5.8	4 months	Singh et al. 2013b
17.	Laboratory Scale (In-activated)^a^	India	Cattle (H/F^b^)	46.6	6 months	Rawatet al. 2014
18	Commercial Scale (In-activated)^a^	India	Cattle (Hariana type)	89.3	9 months	Singh et al. 2015
19	Commercial Scale (In-activated)^a^	India	Goat (Jakhrana)	45.5	12 months	Singh et al. 2017

^a^Indigenous vaccine is now commercialized.

^b^Holstein Friesian breed.

### 2.2. Effects of vaccination on production

Vaccine programs strongly suggested that vaccination in case of JD using vaccine developed from ‘native strain’ was more of a 'therapeutic' than 'preventive' effect, as confirmed by the positive results obtained by vaccinating infected adult animals (Singh et al. 2013; Juste and Perez 2011). Indian vaccination trials also confirmed 'therapeutic nature' of the 'indigenous vaccine' (Singh et al. 2010a, 2013, 2017). Vaccinations not only prevented development of sub-clinical cases into clinical cases but also cured infected adult animals and led to increased production at highly profitable benefit-to-cost ratio (Table 4).

**Table 4.** Effects of vaccination on production parameters (mortality or clinical cases).

Sn	Name/kind of vaccine	Country	Species (breed)	Reduction (%)	Period of study	Reference
1.	Weybridge (Live)	UK	Cattle	99.06	–	Wilesmith, 1982
2.	Lelystad (Killed)	The Netherlands	Cattle	91.82	–	Kaliset al. 1992
3.	Lio-Johne (Live)	Spain	Sheep	78.29	–	Aduriz, 1993
4.	Gudair (Killed)	Australia	Sheep	87.5	–	Windsor et al. 2003
5.	Neoparasec (Live)	New Zealand	Sheep	71.43	One year	Gwozdzet al. 2000
6.	Laboratory Scale (Live)	Greece	Goat	82.78	–	Xenoset al. 1988
7.	Laboratory Scale (In-activated)^a^	India	Goat (Barbari type)	54.8	7 months	Singh et al. 2010a
8.	Laboratory Scale (In-activated)^a^	India	Goat (Jamunapari)	24.6	7 months	Singh et al. 2013
9.	Laboratory Scale (In-activated)^a^	India	Goat (Mehsana)	40.0	4 months	Singh et al. 2013b
10.	Laboratory Scale (In-activated)^a^	India	Cattle (H/F)^b^	95.0	6 months	Rawatet al. 2014
11.	Commercial Scale (In-activated)^a^	India	Goat (Jakhrana)	53.8	12 months	Singh et al. 2017

a Indigenous vaccine is now commercialized.

b Holstein Friesian breed.

### 2.3. Effects of vaccination on histological lesions

Vaccination reverses the immuno-pathologic processes that led to the determined progressive intestinal inflammation responsible for clinical disease in such a way that immunized individuals were able to arrest the progression of infection and ensuing lesions. This resulted in the reduction of the excretion of MAP bacilli and significant decrease in the severity of clinical signs and economic losses (Table 5). According to the 1985 report, vaccination resulted in 98.0% reduction in postmortem finding of lesions, which during the period of 16 years, reduced incidence from 53.0% to 1.0% (Saxegaard and Fodstad 1985; Juste and Perez 2011).'Indigenous vaccine' has been extensively applied in the 4 species of domestic livestock belonging to different breeds, locations and management conditions over period of past 10 years and has shown excellent performance. Vaccination of advance cases of animals suffering from JD infection have come back in to health and regained productive life (Singh et al. 2015; Singh et al. 2017).

**Table 5.** Effects of vaccination on histological lesions (globally).

Sn	Name/kind of vaccine	Country	Species (breed)	Reduction (%)	Period of study	Reference
1	Laboratory Scale (Killed)	The Netherlands	Cattle	58.9	12 years	van Schaik et al. 1996
2	Silirum (Killed)	Spain	Cattle	38.6	–	García-Pariente et al. 2005
3	Laboratory Scale (Killed)	Iceland	Sheep	93.5	–	Sigurdsson,
4	Lio-Johne (Live)	Spain	Sheep	100.0	–	Aduriz, 1993
5	Mycopar (Killed)	USA	Sheep	75.3	–	Thonney and Smith, 2005
6	Gudair (Killed)	Australia	Sheep	72.7	5 years	Reddacliff et al. 2006
7	Gudair (Killed)	New Zealand	Sheep	75.5	16 months	Griffin et al. 2009
8	Laboratory Scale (Live)	Norway	Goat	97.1	14 years	Saxegaard and Fodstad, 1985
9	Gudair (Killed)	Spain	Goat	65.8	–	Corpa et al. 2000
10	Laboratory Scale (In-activated)	USA	Goat	66.6	∼9 months	Kathaperumal et al. 2009
11	Laboratory Scale (In-activated)^a^	India	Goat (Barbari)	75.0	–	Singh et al. 2007
12	Laboratory Scale (In-activated)^a^	India	Goat (Barbari)	57.1	7 months	Singh et al. 2010a
13	Commercial Scale (In-activated)^a^	India	Cattle (Hariana type)	66.7	4 months	Singh et al. 2015

a Indigenous vaccine is now commercialized.

## 3. Next generation vaccines

Many efforts have been made to identify MAP antigens in development of subunit vaccines using genomic and proteomic analysis. Since the production of IFN-γ activated by Th1-mediated immune responses is critical in reducing the number of bacilli in early stages of MAP infection, identifying antigens that prompt strong Th1 responses is essential for the advancement of subunit vaccines (Rosseels and Huygen 2008).

Several antigens/proteins were tested for use as potential vaccine candidates: heat shock protein 70 (Hsp70) (Koets et al. 1999), antigen 85 complex proteins (Ag85A, Ag85B, and Ag85C) (Shin et al. 2005), lipoproteins (LprG and MAP0261c) (Huntley et al. 2005; Rigden et al. 2006), PPE family proteins (MAP1518 and MAP3184) (Nagata et al. 2005), superoxide dismutase (Shin et al. 2005) and alkyl hydroperoxide reductases (AhpC, AhpD) (Olsen et al. 2005). Study by Koets et al. (2006) reported that Hsp70 has been widely used as a subunit vaccine candidate and vaccination using Hsp70 minimize bacterial load as compared with non-vaccinated cattle experimentally challenged with MAP.

Several candidates were evaluated for their ability to induce protective immune responses. However, they were only evaluated in mouse models. Recently, Gupta et al. (2016) have identified 14 immunogenic natural secretory proteins from native ‘S 5’ strain of MAP ‘Indian Bison type’ biotype prevalent in Indian domestic livestock and human population. Diagnostic potential of natural and recombinant immunogenic secretory proteins were evaluated by ‘indirect ELISA’ and working well in diagnosing the active infection of MAP (Chaubey et al. 2018) and will be good candidates to be used as ‘vaccine candidates’ in future.Recently, to improve the competence of the MAP live attenuated vaccines, JDIP research consortium established a three-phase vaccine candidate evaluation method (Bannantine et al. 2014). First phase, a screening test using *in vitro* bovine monocyte-derived macrophage model was conducted by Lamont et al. (2014) to evaluate many live attenuated vaccine candidates constructed until 2014, second phase was a challenge test using the mouse model, and third phase was assessment of protective effects using goat model.

## 4. Commercially available vaccines and future scope

First MAP vaccine consisted of live non-virulent MAP and oil-based adjuvant was developed by Vallee and Rinjardin 1926. Since then, a number of whole-cell killed vaccines, live attenuated and inactivated vaccines were developed to prevent bovine, ovine and caprine JD. Currently, three commercial vaccines (whole cell killed) viz., Mycopar^®^, Gudair^®^ and Silirum^®^ are available (Bastida and Juste 2011). Mycopar^®^ is the only approved vaccine manufactured using MAP ‘strain 18’ (member of the family of *Mycobacterium avium* subspecies paratuberculosis) against bovine JD in the USA (Patton 2011). Gudair^®^ is manufactured by Zoetis (formerly by CZ Veterinaria in Spain) using heat inactivated MAP '316F' strain adjuvanted with mineral oil used in Australia for the control of ovine JD (Windsor 2006). An Australian study opined reduction in the prevalence of MAP shedding after vaccination with their longitudinal study (Eppleston et al. 2005). However, a cross-sectional study by Windsor in 2014 revealed that shedding of MAP persisted in the majority of herds, despite vaccination of lambs. Silirum^®^ consists of MAP ‘316F’, similar to Gudair^®^ and is manufactured by Zoetis AU to prevent bovine JD. Effectiveness of Silirum^®^ vaccine was studied in young farmed deer in New Zealand which revealed that vaccination has reduced the prevalence of clinical JD (Stringer et al. 2013).

Regardless of the advantages of vaccination, one major drawback of 'whole-cell killed' vaccine is interference with diagnostic tests currently used in bovine tuberculosis and paratuberculosis (Kohler et al. 2001; Muskens et al. 2002). Therefore, current research focused towards the development of DIVA test to DIVA (Jayaraman et al. 2016; Chaubey et al. 2018). These vaccines have the potential to produce false positive results in serological tests for paratuberculosis such as ELISA because the commercial ELISA kit consisted of crude MAP antigens, which hinder DIVA (Santema et al. 2011). However, in the IFN-γ assay, stimulation with MAP PPD-B produced strong responses similar to MAP PPD-J (purified protein derivatives) in MAP vaccinated animals (Muskens et al. 2002; Stabel et al. 2011). Because of this cross-reaction with other mycobacteria many countries that are running *M. bovis* eradication programs do not use vaccination policies. However, these problems can be overcome by development of new diagnostic assays using immunogenic natural or recombinant secretory proteins as ‘vaccine candidates’ (Gupta et al. 2016). Another drawback of whole cell killed vaccines is the substantial tissue damage at the injection site and accidental self-inoculation, which may cause serious side-effects (Patterson et al. 1988). However, there is a vaccine adjuvanted with highly refined mineral oils such as Silirum^®^ to decrease the formation of granuloma at the site of injection (Rosseels and Huygen 2008). Therefore, major recommendation which comes from this review is that for a chronic and insidious infection like JD, there cannot be global ‘vaccines' or 'diagnostic kits', the solutions have to come from local 'pathogenic bio-types'/strains of MAP prevalent in particular species of livestock and regions/agro-climatic zones.

## 5. Conclusion

Vaccines against paratuberculosis have been developed by diverse approaches. Most important factors to consider in 'vaccine studies' are the mechanisms related to the host-pathogen interactions and the 'vaccine biotype' used. Much more efforts are needed to understand exactly how bacteria can evade the host defense system, and these should focus on not only an adaptive immune system but also innate immunity. Vaccines that can induce both cellular and humoral immune responses may have improved protective effects. Using local or indigenous strains provide better 'protective index' as compared to vaccines based on foreign strains. Despite some limitations with particular vaccine candidates, vaccines are the most 'effective strategy' to reduce or control or eradicate JD in livestock herds globally. Disease being highly endemic in developing and less developed countries, as suggested by low per animal productivity and in view of the limitations of resources, vaccines are the only cost effective methodology for the management of ‘incurable’ JD at country/regional level.
